# Health Literacy and Quality of Life in Young Adults From The Belgian Crohn's Disease Registry Compared to Type 1 Diabetes Mellitus

**DOI:** 10.3389/fped.2021.624416

**Published:** 2021-02-05

**Authors:** Constance Carels, Lucas Wauters, An Outtier, Filip Baert, Peter Bossuyt, Arnaud Colard, Danny De Looze, Marc Ferrante, Alexander Goegebuer, Bruno Hauser, Robert Hilbrands, Ilse Hoffman, Bart Keymeulen, Isabelle Paquot, Isabelle Ruytjens, Marc Simoens, Clara Thienpont, Annelies Verreth, Bram Verstockt, Séverine Vermeire, Gigi Veereman

**Affiliations:** ^1^Pediatric Gastroenterology, UZ Brussels, Jette, Belgium; ^2^Gastroenterology and Hepatology, University Hospitals, KU Leuven, Leuven, Belgium; ^3^Gastroenterology, AZ Delta, Roeselare, Belgium; ^4^Gastroenterology, Imelda Ziekenhuis, Bonheiden, Belgium; ^5^Gastroenterology, CHC Liège, Liège, Belgium; ^6^Gastroenterology and Hepatology, UZ Gent, Ghent, Belgium; ^7^Gastroenterology, Heilig Hart Ziekenhuis Leuven, Leuven, Belgium; ^8^Diabetes Clinic, UZ Brussel, Jette, Belgium; ^9^Pediatric Gastroenterology, University Hospitals, KU Leuven, Leuven, Belgium; ^10^Pediatric Gastroenterology, CHC Liège, Liège, Belgium; ^11^Gastroenterology, ZNA Middelheim, Antwerp, Belgium; ^12^Gastroenterology, ZNA Jan Palfijn, Merksem, Belgium; ^13^Gastroenterology, ZNA Stuivenberg, Antwerp, Belgium; ^14^Gastroenterology, AZ Sint-Jozef, Malle, Belgium

**Keywords:** health literacy, quality of life, Crohn's disease, BELCRO registry, type 1 diabetes mellitus

## Abstract

**Background and Aims:** The management of chronic inflammatory bowel diseases in youth is challenging. We aimed to determine health literacy (HL), quality of life (QoL) and clinical outcomes in young adults from the BELgian CROhn's disease registry (BELCRO) in comparison to type 1 diabetes mellitus (DM) as a control.

**Methods:** In this prospective and observational study, young adults with Crohn's disease (CD) diagnosed < 18 years and with > 5 years disease duration and a comparable group of patients with DM completed validated HL, QoL and work productivity and activity impairment questionnaires (HLS-EU-Q16, EQ-5D-5L and WPAI). HL was scored as sufficient (13–16), problematic (9–12) or inadequate (0–8). QoL was dichotomized into “no problems” (EQ-5D level 1) or “problems” (EQ-5D levels 2 to 5). Non-parametric (Mann-Whitney *U*) analyses and Spearman correlations were performed.

**Results:** A total of 52 CD (median [IQR] age of 25.0 [23.8-27.0], 64% male) and 50 DM (age 20.0 [19.0-22.0], 50% male) patients were included. HL was 14.0 [11.0-16.0] for CD and 14.0 [11.3-14.8] for DM (*p* = 0.6) with similar proportions of sufficient (60 vs. 68%, *p* = 0.4), problematic (34 vs. 26%, *p* = 0.3) and inadequate HL (both 6%, *p* = 1). Although QoL was comparable for CD and DM (77.0 [68.8-82.0] vs. 75.0 [65.0-80.0] %, *p* =0.4), CD had a trend for higher pain/discomfort (50 vs. 32%, *p* = 0.06). HL and QoL correlated in CD (*r* = 0.6, *p* < 0.001) and DM patients (*r* = 0.6, *p* < 0.001). Fewer CD patients with recent hospitalization/surgery had sufficient HL (31 vs. 69%, *p* = 0.01) and had lower QoL (70.0 [60.0-77.0] vs. 80.0 [70.0-85.0], *p* = 0.04) compared to those without.

**Conclusions:** Selected young Belgian adults suffering from CD for >5 years have similar and sufficient HL compared to DM patients. However, CD patients requiring hospitalization/surgery have lower HL, which indicates the need for targeted educational programs.

## Introduction

The care for patients with chronic inflammatory diseases is one of the major challenges in today's society. Crohn's disease (CD) is an inflammatory bowel disease (IBD) which has important repercussions on daily life (1–3). Many patients are affected in their youth, which impacts educational and professional choices. Huang et al. (4) state that “to prepare for the transition from pediatric to adult-oriented health care, adolescents must develop the ability to obtain, process and understand basic health information, make appropriate health decisions and interact effectively with health care professionals”. This ability is called Health Literacy (HL) (4). Guidelines for transition have been proposed (5, 6). However, physicians do not routinely use validated tools to determine the patients' “health literacy-related readiness” for this transition in IBD (4). Moreover, a higher proportion of non-participation in the work force can be found in IBD patients with a tendency for more sick leaves, especially after surgery (7). CD appears to have higher repercussions on work force participation than ulcerative colitis (UC), and these consequences are more frequently observed in younger patients as well as those with a higher education level (3).

Similar to CD, patients with type 1 (juvenile–onset) diabetes mellitus (DM) with poor HL encounter difficulties to process and understand health information regarding their disease (8). Diabetics with poor health behaviors are at greater risk for numerous adverse health outcomes (9, 10). A study on HL showed that despite the belief of disease control in most DM patients, this was not the case in reality (11). Patients who incorrectly believed that they could manage their disease were less likely to adapt their lifestyle to enhance self-control of glucose levels (11). On the other hand, DM patients with higher HL controlled their glycated hemoglobin (HbA_1_c) levels better and were less likely to smoke (10). Another study emphasized that HL empowered the individual to manage the disease (12).

Prospective long-term registries are necessary to provide insights by monitoring disease and treatment patterns. Moreover, pediatric registries provide the unique opportunity to study adolescents during their transition to adulthood. In May 2008, the prospective pediatric (diagnosis prior to their 18th birthday) BELgian CROhn's disease registry (BELCRO) was initiated in order to study the disease presentation and phenotype of both previously and newly diagnosed children and adolescents with CD in Belgium (13). Treatment and outcomes after 3- and 5-year follow-up (FU) have previously been published (14, 15). Although the 5-year FU data from the BELCRO demonstrated a satisfactory long-term outcome with anti-TNF therapy in over two thirds of patients (15), the relation between disease activity and HL, quality of life (QoL), work productivity impairment (WPI), and activity impairment (AI) remains unknown.

HL and QoL of Belgian CD patients have not been prospectively assessed. Hence, the objectives of this study were to assess the HL, QoL, WPI, and AI of young Belgian adults who developed CD during their childhood and were followed in the BELCRO registry, compared to young diabetics. In Belgium there are organized clinics with multi-disciplinary care and education for children and adolescents with DM (so called “medische conventies”). These facilities are so far lacking for young patients with CD. Therefore, at present adolescents with CD cannot benefit from structured educational programs. Furthermore, HL was studied across different levels of QoL-related domains and level of education or employment. Finally, the relationship between HL, QoL, WPI, and AI with patient characteristics and clinical outcomes, including hospitalizations and surgery in CD patients, was investigated.

## Methods

### Study Design and Protocol

The study protocol of this prospective and observational study was approved by the Institutional Review Board (IRB) ZNA Middelheim (B00920083829), after approval by the board of the Belgian Society for Pediatric Gastroenterology, Hepatology and Nutrition (BESPGHAN) and the Scientific Committee of the Belgian Inflammatory Bowel Disease Research and Development group (BIRD). The BELCRO registered patient data from April 2008 until 2015. Both previously and newly diagnosed patients were included during a registration period of 2 years (May 1st, 2008 to April 30th, 2010) with subsequent 5 years FU. Disease course, treatment and outcomes were monitored through collaboration between pediatric and adult specialists from 11 centers of BESPGHAN and 12 centers of BIRD (14). The FU study was conducted according to the declaration of Helsinki and Good Clinical Practice guidelines. Informed consent was obtained from patients since all subjects had reached 18 years of age.

### Subject Enrolment

Subjects from 2 populations were included: (1) CD patients from the BELCRO-cohort and (2) type 1 DM patients, as a control group.

#### CD Patients

Patients previously included in the BELCRO with > 5 years FU who had reached the age of 18 were contacted through the adult centers where the care had been transferred. The choice of 5 years FU was based on the fact that by then, most CD patients had transitioned to adult care. One of the lead researchers contacted and requested the treating physician to invite the patient to participate in the FU study at the next planned clinic visit. After obtaining informed consent, the treating physician completed the disease assessment including Crohn's Disease Activity Index (CDAI), Body Mass Index (BMI), current treatment, hospitalizations and/or surgery during the last 3 years, which was decided by the investigators. The patient completed the remaining clinical and additional psychosocial variables including education level (lower or higher education) and current occupation.

#### DM Patients

Diabetic patients aged 18 to 28 years old were contacted during their clinic visit at the Diabetes Clinic in UZ Brussels by one of the lead researchers. After the informed consent form was signed, the lead researcher completed the disease assessment including HbA_1_c results, complications or co-morbidities. The remaining clinical and additional psychosocial variables including education level and current occupation were filled out by the patient.

### Study Procedures

Similar tools for the evaluation of HL, QoL, WPI and AI (Dutch and French) were used in both groups:

#### Health Literacy

The 16-item Dutch and French version of the European Health Literacy Survey Questionnaire (HLS-EU-Q) were used (16), with scores between 0 and 8 indicating inadequate HL, scores between 9 and 12 problematic HL, and scores between 13 and 16 sufficient HL (17).

#### Quality of Life

The Dutch and French versions of the EuroQol standardized instrument or EQ-5D-5L were used (18), with 5 levels for health status regarding problems with mobility, self-care, normal activities, pain/discomfort or anxiety, ranging from “no problems” to “extreme problems.” The last question was the EQ Visual Analog Scale (VAS), which measured the self-rated health of the patient on the day of study participation ranging from 0 (meaning “the worst health you can imagine”) to 100 (which stands for “the best health you can imagine”).

#### Work and Activity Impairment

For CD patients already employed, the Dutch and French versions of the WPAI Questionnaire for CD were used, which assessed the number of hours missed from work due to health problems and impact on productivity or normal activities (19). For DM patients who were already employed, the Dutch or French version of the WPAI Questionnaire for general health (GH) was filled out, since there is no specific WPAI for DM (19).

### Data Collection and Analysis

QoL was dichotomized into “no problems” (EQ-5D level 1) or “problems” (EQ-5D levels 2 to 5) (20). Work productivity and activity impairment were scored as percentages. Continuous variables were summarized as medians with interquartile ranges (IQR) and categorical data as percentages. Non-parametric (Mann-Whitney U) analyses were used to compare demographic and clinical variables between CD and DM patients and within subgroups. Non-parametric correlations (Spearman) were performed between variables of interest. Two-tailed *P* values with a *P* value of < 0.05 were used in all analyses to determine statistical significance. Statistical analyses were performed with Prism version 8 (GraphPad Software, San Diego, USA).

## Results

### Patient Flow

**Figure 1:**
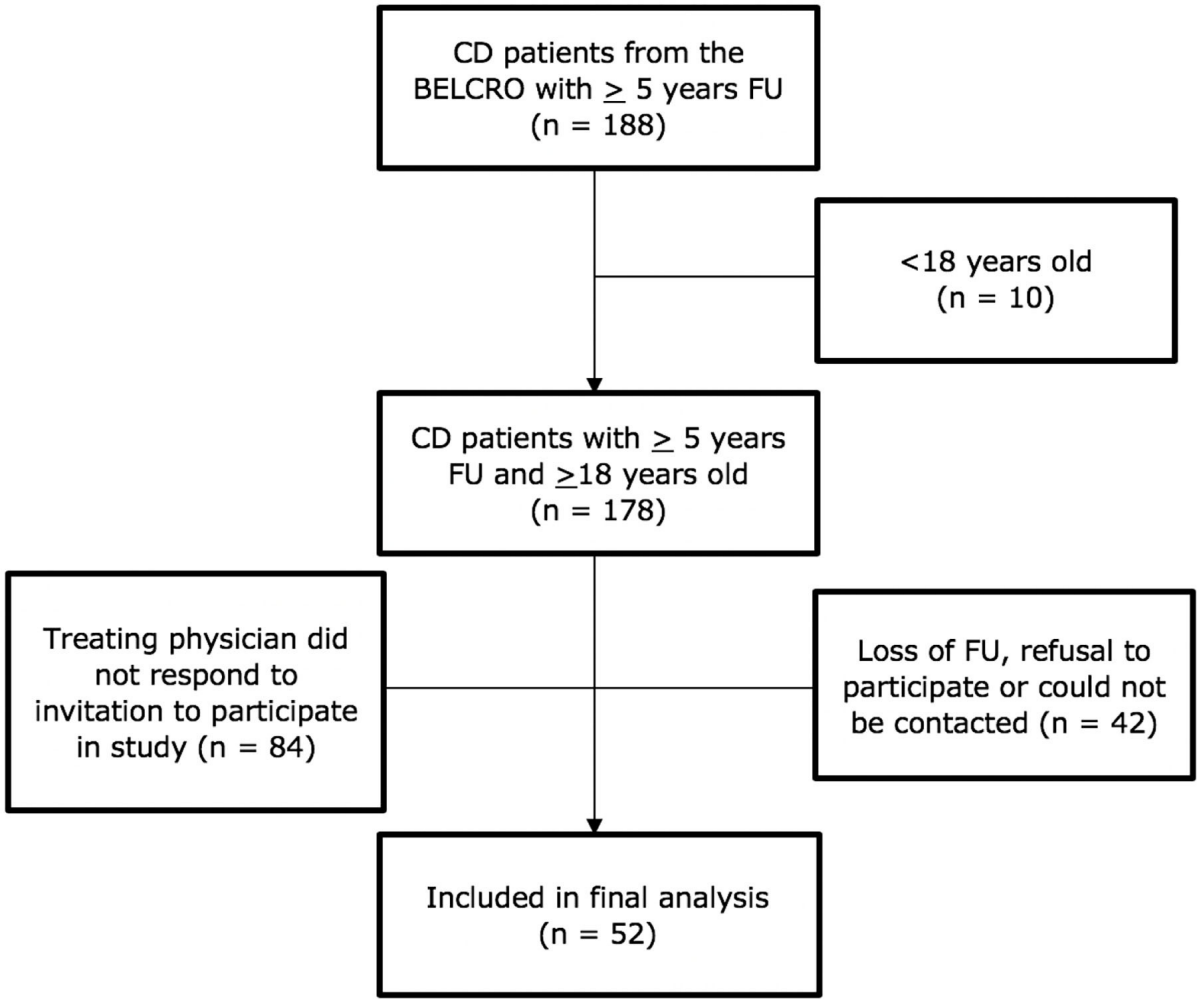
Patient flowchart for CD. BELCRO, BELgian pediatric CROhn's Disease Registry; CD, Crohn's Disease; FU, Follow-up.

As shown in Figure 1, 188 CD patients included in the BELCRO registry had at least 5 years FU. Ten patients were under 18 years of age and therefore excluded. Of the remaining 178 young adult CD patients with 5 years FU, 84 patients could not be reached as the treating physician did not respond to the invitation to participate in this FU study. Forty-two patients could not be included due to loss of FU, refusal to participate or because they could not be reached by their treating physician. In the final analysis, 52 CD patients with at least 5 years FU were included from different centers across Belgium. Demographics were similar for CD patients included vs. excluded from the study. For the control group, 50 type 1 DM patients between 18 and 28 years old were included from one center (UZ Brussels, Belgium) by one of the lead researchers. Out of the 50 DM patients, only 6 had a <5-year FU because they were diagnosed <5 years ago.

### Patient Characteristics

Baseline characteristics of CD (*n* = 52, 64% male) and DM (*n* = 50, 50% male) patients are shown in Table 1. CD patients were older in comparison with the DM patients, with a median [IQR] age of 25.0 [23.8–27.0] and 20.0 [19.0–22.0] years (*p* < 0.0001), respectively. The eldest patients of the cohort were 28 years old. The median CDAI value of 51 CD patients was 76.5 [20.2–135.9]. Median HbA_1_c for DM was 7.9 [7.1–8.8] %. BMI was significantly lower for CD compared to DM patients with a median of 23.1 [20.0–25.1] compared to 25.5 [21.8–28.4] (*p* = 0.02). There were fewer smokers in the CD group compared to DM patients (13.5 vs. 42%, *p* = 0.001). Significantly more CD patients attended higher education than DM patients (65 vs. 6%, *p* < 0.0001) and more CD patients than DM patients were employed at the time of their participation (76 vs. 34%, *p* < 0.0001).

**Table 1:** Baseline characteristics of CD and DM patients.

	**CD (*n* = 52)**	**DM (*n* = 50)**	***P* value**
Median age (IQR), years	25.0 (23.8–27.0)	20.0 (19.0–22.0)	**<** **0.0001**
Male gender (%)	33 (64)	25 (50)	0.2
Median CDAI (IQR)^*^	76.5 (20.2–135.9)	/	/
Median HbA_1_c (%)	/	7.9 (7.1–8.8)	/
Median BMI (IQR), kg/m^2^	23.1 (20.0–25.1)	25.5 (21.8–28.4)	**0.02**
Active or ex-smoker (%)	7 (13.5)	21 (42)	**0.001**
Lower level of education (high school)	18 (35)	32 (64)	**0.003**
Higher level of education (Study Bachelor, Master or University College) (%)^*^	33 (65)	3 (6)	**<0.0001**
Current occupation (%)^*^	39 (76)	17 (34)	**<0.0001**

* *1 patient missing due to missing values*.

*BMI, Body Mass Index; CD, Crohn's Disease; CDAI, Crohn's Disease Activity Index; DM, Diabetes Mellitus; HbA1c, Glycated hemoglobin; IQR, Interquartile Range*.

*Statistically significant values in bold*.

### Study Questionnaires

Comparison of the questionnaires' variables, consisting of HL, QoL, WPI and AI, are summarized in Table 2.

**Table 2:** Comparison of the questionnaires' variables between CD and DM patients.

	**CD (*n* = 52)**	**DM (*n* = 50)**	***P* value**
Median HL (IQR)	14.0 (11.0–16.0)	14.0 (11.3–14.8)	0.6
Sufficient (%)	31 (60)	34 (68)	0.4
Problematic (%)	18 (34)	13 (26)	0.3
Inadequate (%)	3 (6)	3 (6)	1
Median QoL (IQR), %	77.0 (68.8–82)	75.0 (65.0–80.0)	0.4
Problems with:			
Mobility (%)	7 (13)	8 (16)	0.7
Self-care (%)	1 (2)	0 (0)	0.3
Activities (%)	21 (40)	14 (28)	0.2
Pain/Discomfort (%)	26 (50)	16 (32)	0.06
Anxiety (%)	20 (38)	20 (40)	0.9
Median WPI (IQR)^*^	0.1 (0.0–0.3)	0.2 (0.1–0.4)	0.06
Median AI (IQR)	0.2 (0.0–0.4)	0.2 (0.1–0.4)	0.9

* *Data for 38 CD (2 patients missing due to missing values) and 25 DM patients*.

*AI, Activity Impairment; CD, Crohn's Disease; DM, Diabetes Mellitus; HL, Health Literacy; IQR, Interquartile Range; QoL, Quality of Life; WPI, Work Productivity Impairment*.

#### Health Literacy

Median HL was similar for both CD and DM (14.0 [11.0–16.0] vs. 14.0 [11.3–14.8], *p* = 0.6). Equal proportions of sufficient (60 vs. 68%, *p* = 0.4), problematic (34 vs. 26%, *p* = 0.3) and inadequate HL (both 6%, *p* = 1) were observed for CD and DM, respectively.

#### Quality of Life

Median QoL was comparable for CD and DM (77.0 [68.8–82.0] vs. 75.0 [65.0–80.0] %, *p* = 0.4) with a trend for higher pain/discomfort (50 vs. 32%, *p* = 0.06) for CD patients. While only a minority of CD and DM patients had problems with mobility and only one CD patient with self-care, problems with usual activities, pain/discomfort and anxiety were more common but similar in both groups (all *p* > 0.05). Comparison of CD and DM patients with and without problems in their usual activities, pain/discomfort and anxiety is shown in Table 3.

**Table 3:** Comparison of CD and DM patients with or without problems in usual activities, pain/discomfort and anxiety.

	**CD (*****n*** **=** **52)**			**DM (*****n*** **=** **50)**		
**Usual activities**	**Problems (*n* = 21)**	**No problems (*n* = 31)**	***P* value**	**Problems (*n* = 14)**	**No problems (*n* = 36)**	***P* value**
Median HL (IQR)	11.0 (10.0–13.5)	15.0 (13.0–16.0)	**0.0006**	12.5 (11.0–14.0)	14.0 (12.8–15.0)	0.1
Median QoL (IQR), %	65.0 (50.0–70.5)	80.0 (77.0–90.0)	**<0.0001**	65.0 (46.3–74.8)	75.5 (70.0–80.0)	**0.02**
Median CDAI (IQR)^*^	131.1 (95.0–232.3)	36.6 (13.0–78.6)	**0.001**	/	/	/
**Pain/Discomfort**	**Problems (*****n*** **=** **26)**	**No problems (*****n*** **=** **26)**	***P*** **value**	**Problems (*****n*** **=** **16)**	**No problems (*****n*** **=** **34)**	***P*** **value**
Median HL (IQR)	12.5 (10.3–14.8)	15.0 (12.0–16.0)	**0.02**	11.5 (10.8–14.0)	14.0 (13.0–15.0)	**0.01**
Median QoL (IQR), %	70.0 (60.0–80.0)	80 (75.5–90)	**<0.0001**	68.0 (50.0–77.0)	75.0 (70.0–80.0)	0.05
Median CDAI (IQR)^**^	123.6 (89.1–232.3)	20.9 (0.0–41.5)	**<0.0001**	/	/	/
**Anxiety**	**Problems (*****n*** **=** **20)**	**No problems (*****n*** **=** **32)**	***P*** **value**	**Problems (*****n*** **=** **20)**	**No problems (*****n*** **=** **30)**	***P*** **value**
Median HL (IQR)	12.0 (10.0–14.0)	15.0 (11.8–16.0)	**0.006**	13.5 (11.0–14.0)	14.0 (13.0–15.0)	0.2
Median QoL (IQR), %	70.0 (52.5–75.0)	80.0 (75.0–88.8)	**0.0006**	67.5 (57.5–75.3)	78.5 (70.0–80.0)	0.1
Median CDAI (IQR)^***^	119.1 (56.1–246.2)	38.5 (15.6–108.3)	**0.03**	/	/	/

* *Data for 20 CD patients with problems (1 missing)*.

** *Data for 25 CD patients with problems (1 missing)*.

*** *Data for 19 CD patients with problems (1 missing)*.

*CD, Crohn's Disease; CDAI, Crohn's Disease Activity Index; DM, Diabetes Mellitus; HL, Health Literacy; IQR, Interquartile Range; QoL, Quality of Life*.

*Statistically significant values in bold*.

When comparing CD patients with and without issues in their usual activities, we found that CD patients encountering difficulties had significantly lower HL scores compared to those not (*p* = 0.0006). Similar results could be observed regarding their QoL (*p* < 0.0001). In addition, CD patients who faced more difficulties in their usual activities had a higher median CDAI compared to those who did not (*p* = 0.001). On the other hand, DM patients who reported having difficulties to perform their usual activities had a numerically lower HL compared to those who did not (*p* = 0.1). The median QoL was significantly lower for DM patients facing difficulties vs. those who did not (*p* = 0.02).

CD patients who reported feeling pain/discomfort had a significantly lower median HL (12.5 [10.3–14.8] vs. 15.0 [12.0–16.0], *p* = 0.02) and median QoL (70.0 [60.0–80.0] vs. 80.0 [75.5–90.0] %, *p* < 0.0001) compared to those who did not. The median CDAI was significantly higher in patients reporting pain/discomfort (123.6 [89.1–232.3] vs. 20.9 [0.0–41.5], *p* < 0.0001). Similarly, DM patients experiencing pain/discomfort had a significant lower median HL (11.5 [10.8–14.0] vs. 14.0 [13.0–15.0], *p* = 0.01) and a trend for a lower median QoL (68.0 [50.0–77.0] vs. 75.0 [70.0–80.0]%, *p* = 0.05) compared to those not experiencing any pain/discomfort.

Regarding anxiety, the median CDAI value was higher for anxious CD patients in comparison with non-anxious CD patients (119.1 [56.1–246.2] vs. 38.5 [15.6–108.3], *p* = 0.03). The median HL (12.0 [10.0–14.0] vs. 15.0 [11.8–16.0], *p* = 0.006) and median QoL (70.0 [52.5–75.0] vs. 80.0 [75.0–88.8] %, *p* = 0.0006) were significantly lower for CD patients with anxiety versus those without. DM patients with anxiety had a similar HL compared those without (*p* = 0.2). The median QoL was lower in those with anxiety vs. those without [67.5 (57.5–75.3) vs. 78.5 (70.0–80.0) %, *p* = 0.1].

#### Work Productivity and Activity Impairment

Subjects registered “work” as regular employment or temporary student jobs. There was a numerical though not significant lower WPI in CD patients who were working at the time of participation compared to DM (10 [0–30] vs. 20 [10–40] %, *p* = 0.06). The median AI due to their disease was similar (*p* = 0.9). CD patients who were working had a similar HL (14.5 [11.0–16.0] vs. 13.0 [11.0–14.3], *p* = 0.2) and QoL (80.0 [70.0–80.5] vs. 74.0 [63.8–86.3] %, *p* = 0.7) compared to those not working. Working DM patients had similar HL (13.5 [12.0–14.0] vs. 14.0 [11.0–15.0], *p* = 0.5) and QoL (75.0 [65.0–80.0] vs. 70.5 [65.0–80.0] %, *p* = 0.9) compared to those who were not working. CDAI for CD workers was 61.8 [20.2–125.2] compared to 105.9 [29.0–269.3] for the non-workers (*p* = 1). When comparing working CD and DM participants, both groups had a similar HL (14.5 [11.0–16.0] vs. 13.5 [12.0–14.0], *p* = 0.2) and QoL (80.0 [70.0–80.5] vs. 75.0 [65.0–80.0], *p* = 0.3).

### Clinical Outcomes

#### Disease Activity

At the time of assessment, 39 CD patients were in clinical remission (CDAI <150) and 12 CD patients had active disease (median CDAI 251.8 [205.9–307.0], maximum value of 415.6). CD patients with active disease had a significant lower median HL (11.5 [10.0–13.3] vs. 15.0 [11.5–16.0], *p* = 0.02) and lower QoL (67.5 [56.3–80.0] vs. 80.0 [70.0–85.0], *p* = 0.03) compared to the CD patients in remission.

For DM patients, disease activity was evaluated by their HbA_1_c values. HbA_1_c is dependent on age. Young adults should reach < 7.0% since little comorbidity exists (21). Median HbA_1_c of all DM patients was 7.9 [7.1–8.8] %. Out of the 50 DM patients, 41 had a median HbA_1_c value of 7% or higher, while 9 patients reached the HbA_1_c goal of < 7%.

#### Hospitalization/Surgery

During the last 3 years counting from the date of study participation, 39 out of the 52 CD patients did not need hospital admission for their disease (median days spent in a hospital 0.0 [0.0–0.5]). Thirteen out of the 52 CD patients with median age of 26.0 [25.0–27.0] years required hospitalization and/or surgery in the course of the last 3 years (Table 4). They had a higher but not significantly different median CDAI (105.9 [47.7–246.2] vs. 48.8 [19.3–125.8], *p* = 0.1), with a trend for lower HL (12.0 [11.0–13.0] vs. 15.0 [11.0–16.0], *p* = 0.1) and a significantly lower QoL (70.0 [60.0–77.0] vs. 80.0 [70.0–85.0], *p* = 0.04) compared to patients who did not need hospitalization and/or surgery. The proportion of sufficient HL was significantly lower for CD patients with recent hospitalization and/or surgery compared to those without (31 vs. 69%, *p* = 0.01). Conversely, the proportion of problematic HL was significantly higher in the group with hospitalization in comparison with the other group (61 vs. 26%, *p* = 0.02). CD patients who were recently hospitalized reported to have numerically more WPI and AI due to their condition compared to those who did not (*p* = 0.3 and *p* = 0.2, respectively).

**Table 4:** Comparison between CD patients with and without hospitalization/surgery.

**Hospitalization/surgery**	**Yes (*n* = 13)**	**No (*n* = 39)**	***P* value**
Median age (IQR)	26.0 (25.0–27.0)	25.0 (23.5–26.5)	0.5
Median BMI (IQR)	22.1 (19.0–24.6)	23.5 (20.5–25.4)	0.3
Median CDAI (IQR)^*^	105.9 (47.7–246.2)	48.8 (19.3–125.8)	0.1
Median HL (IQR)	12.0 (11.0–13.0)	15.0 (11.0–16.0)	0.1
Sufficient (%)	4 (31)	27 (69)	**0.01**
Problematic (%)	8 (61)	10 (26)	**0.02**
Inadequate (%)	1 (8)	2 (5)	0.7
Median QoL (IQR)	70.0 (60.0–77.0)	80.0 (70.0–85.0)	**0.04**
Problems with:			
Mobility (%)	2 (15)	5 (13)	0.8
Self-care (%)	1 (8)	0 (0)	0.08
Activity (%)	7 (54)	14 (36)	0.3
Pain/Discomfort (%)	7 (54)	19 (49)	0.7
Anxiety (%)	6 (46)	14 (36)	0.5
Median WPI (IQR)^**^	0.2 (0.0–0.53)	0.1 (0.0–0.2)	0.3
Median AI (IQR)	0.3 (0.1–0.7)	0.2 (0.0–0.3)	0.2

* *Data for 38 CD patients without hospitalization, 1 patient missing due to missing values*.

** *Data for 8 CD patients with hospitalization (1 patient missing due to missing values) and 29 without hospitalization (1 missing patient due to missing values)*.

*AI, Activity Impairment; BMI, Body Mass Index; CD, Crohn's Disease; CDAI, Crohn's Disease Activity Index; HL, Health Literacy; IQR, Interquartile Range; QoL, Quality of Life; WPI, Work Productivity Impairment*.

*Statistically significant values in bold*.

### Correlations

**Figure 2:**
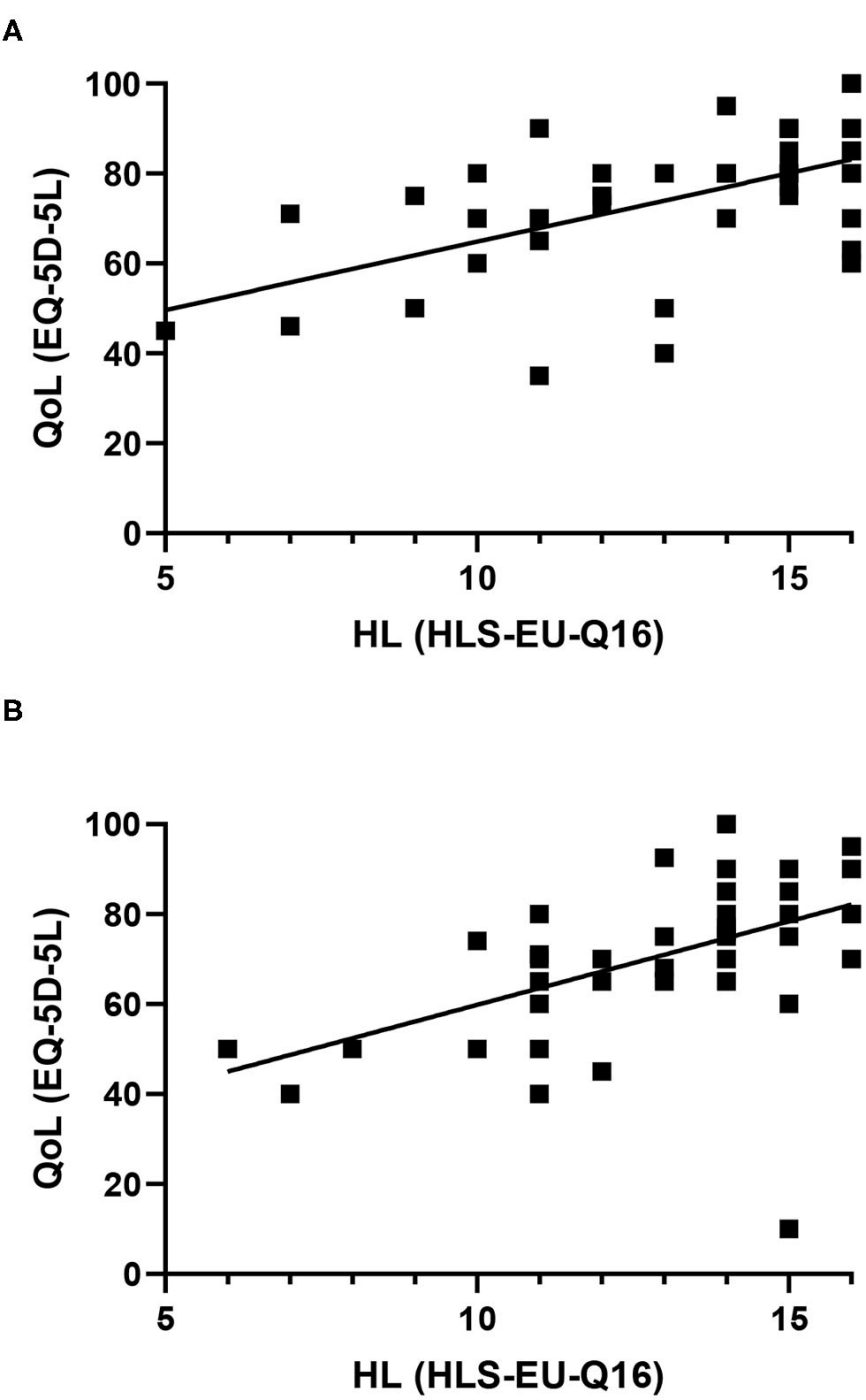
Correlation between health literacy and quality of life in Crohn's disease **(A)** and Diabetes mellitus **(B)** patients.

HL correlated positively with QoL in both CD (*r* = 0.6, *p* = 0) and DM (*r* = 0.6, *p* < 0.0001) patients (Figure 2, Table 5). In addition, HL correlated negatively with WPI for CD (*r* = −0.5, *p* = 0.005) and DM (*r* = −0.2, *p* = 0.3) and with AI for CD (*r* = −0.4, *p* = 0.002) but not DM (r = −0.2, *p* = 0.2) patients. A trend for a negative correlation was found between HL and CDAI in CD (*r* = −0.3, *p* = 0.06) with a negative correlation between CDAI and QoL (*r* = −0.4, *p* = 0.001). Also, WPI (*r* = 0.5, *p* = 0.003) and AI (*r* = 0.5, *p* =0. 001) were significantly correlated with CDAI. In contrast, HbA_1_c did not correlate with HL (*r* = 0.007, *p* = 1), WPI (*r* = 0.08, *p* = 0.7), or AI (*r* = 0.1, *p* = 0.5) in DM patients. However, there was a significant negative correlation between QoL and HbA_1_c (*r* = −0.4, *p* = 0.002).

**Table 5:** Correlations between HL and demographic variables in CD and DM patients.

	**CD (*****n*** **=** **52)**		**DM (*****n*** **=** **50)**	
	**R value**	***P* value**	**R value**	***P* value**
HL–QoL	0.6	**<** **0.0001**	0.6	**<** **0.0001**
HL–AI	−0.4	**0.002**	−0.2	0.2
HL–CDAI^*^	−0.3	0.06	/	/
QoL–CDAI^*^	−0.4	**0.001**	/	/
AI–CDAI^*^	0.5	**0.001**	/	/
HL–HbA_1_c	/	/	0.005	1
QoL–HbA_1_c	/	/	−0.4	**0.002**
AI–HbA_1_c	/	/	0.1	0.5

* *Data for 51 CD patients (1 missing)*.

*AI, Activity Impairment; BMI, Body Mass Index; CD, Crohn's Disease; CDAI, Crohn's Disease Activity Index; DM, Diabetes Mellitus; HbA_1_c, Glycated hemoglobin; HL, Health Literacy; QoL, Quality of Life; WPI, Work Productivity Impairment; #, number*.

*Statistically significant values in bold*.

## Discussion

This is the first study assessing HL, QoL, WPI, and AI (including level of education and employment rates) of young Belgian adult CD patients with at least 5 years FU. The findings were compared with a control group of type 1 DM patients. Although the results show a comparable HL and QoL for CD and DM patients, problems with the level of pain/discomfort were more common in CD. Besides, CD patients with active disease or recent hospitalization or surgery have lower HL and QoL compared to those without. While median HL appeared sufficient for both CD and DM patients, the proportions of inadequate and problematic HL were similar. These are encouraging results since no structured educational programs for young CD patients exist as they do for DM patients (22–24). Interestingly, CD patients facing difficulties in their usual activities, pain/discomfort or anxiety had a significantly lower median HL and QoL and a higher CDAI compared to patients without those issues. Similarly, patients with recent hospitalization or surgery had a higher CDAI and a problematic HL, while those without had sufficient HL although the number of hospitalized patients was small. Median QoL was also lower and WPI higher for hospitalized patients. These findings suggest that improving HL with adequate programs may indeed offer health benefits.

It may seem intuitive that better HL is reflected in better QoL. However, this had not yet been demonstrated and the opposite may also be argued: more knowledge about the disease and possible complications may generate more anxiety. Thus, the correlation between HL and QoL does not prove causality and further prospective studies are needed.

The higher number of CD patients with a higher level of education and employment compared to DM may be explained by the slightly older age in CD. Nevertheless, there was a trend for less WPI for the working CD patients compared to working DM patients which should be confirmed in larger studies. The rate of AI was similar in both groups and although most CD patients were in remission, most DM patients did not reach the targeted HbA_1_c level of < 7.0%. Similar outcomes for the DM patients were found in the 2019 publication of Foster et al. (25) reporting that only a minority of US patients from the “type 1 Diabetes Exchange clinic registry” across all age groups achieved the goal of an HbA_1_c level of <7%. CD patients had a lower BMI than DM patients with a median BMI > 25 kg/m^2^ suggesting an increased proportion of DM patients with overweight as defined by the World Health Organization (WHO) (26). An important increase in BMI in Belgian children with type 1 DM over the last decades has been reported (27).

The relationship between HL, QoL, and CDAI opens novel perspectives in patient management strategies. Although aggressive disease could prevent patient education, the consequence of higher HL including improved QoL and potentially disease control should be studied further. Our findings confirm earlier reports of more active disease in CD patients with limited HL (28). The main differences with our study were the recruitment of adult patients (mean age 47 years) with both CD and UC from only one center and different questionnaires (Newest Vital Sign, the 10-question Short IBD Questionnaire and the Harvey-Bradshaw Index for CD and the Simple Clinical Colitis Activity Index for UC). In a study by Malhotra et al. (29), HL of IBD patients with mean age of 36.1 years (range 18–81 years) was significantly higher than the score attributed by the physician, suggesting that physicians tend to underestimate their patient's HL. However, it is challenging to compare studies that measure HL, especially regarding the multiplicity of different tools (30).

Olesen et al. (31) studied the association between HL and glycemic control in 1,399 Danes with type 1 DM, showing that patients with higher HL levels had a lower HbA_1_c. According to a Turkish study by Esen et al. (32), type 1 DM patients with problematic and insufficient HL, measured with the HLS-EU-Q47, had significantly higher HbA_1_c levels. In a systematic review, Berkman et al. (33) concluded that low HL was associated with less health-related knowledge, poorer health outcomes and use of health care services in general. Vandenbosch et al. (17) investigated whether low HL, measured with the 16-item HLS-EU-Q questionnaire, was linked to greater use of health care services in Belgium. As this study suggested that individuals with low HL levels generally use health care services more often than those with higher HL levels, the correct use of health care services could be improved by increasing the level of HL of a population. Measures to improve HL would thus benefit the health status and outcomes of a population. Regarding QoL, König et al. (34) demonstrated the validity, reliability and responsiveness of the EuroQol EQ-5D questionnaire in IBD and concluded that the EQ-5D can be used to measure QoL in IBD patients. Complications and poor glycemic control in type 1 DM patients also affect negatively QoL, as described by Smith-Palmer et al. (35) in a systematic review.

As already mentioned, Huang et al. (4) evaluated the “HL-related readiness” for transition to adult care in pediatric IBD patients. They demonstrated that health care providers tend to overestimate the “HL-related readiness” for transition. The next step for improving HL in young adult CD patients would be to implement a structured transition program. In 2011, Philpott et al. (36) discussed, among other topics, the different stages of transitional care, including the role of the physicians as well as of the patients. This program should ideally start from the time of diagnosis of CD such that transition can be initiated in early adolescence (6, 36). A transition intervention should be implemented that helps patients understand more about their health and empowers them in their own care, as their education and self-efficacy increases. Self-efficacy is described as “the ability to organize and implement a pattern of behavior necessary for health promotion.” This includes “the ability to monitor symptoms and report them to a health-care professional, to manage their medication and maintain adherence to the prescribed regime, to recognize and understand how to handle a disease flare, and to work in partnership with health care providers” (6). As mentioned by Abraham and Kahn, managing the transition process of IBD patients requires specific attention, communication and careful planning (37). The present study highlights the potential use of disease-specific and general tools during targeted educational programs and especially transition.

There are several limitations to this study. CD patients came from different centers across Belgium while all the patients from the control group were recruited from one center in Brussels. This could have an impact on the results as we compared data from a national level over one center. The dropout rate was considerable for BELCRO patients despite considerable efforts and an excellent collaboration between pediatricians (BELCRO investigators from BeSPGHAN) and internists from the BIRD. However, the BELCRO is unique for Belgium. The study reflects a real-life situation. It may be assumed that patients with higher education were more likely to participate and thereby create a bias: participating patients may have a better HL than the ones we did not reach. This reinforces the need for educational programs. A more objective marker of disease activity was used for DM patients, namely HbA_1_c, in contrast to the CDAI used for CD patients. We did not include psychological distress, which could also influence the studied outcomes. Moreover, no subanalyses were done based on disease location or behavior, biochemical or endoscopic inflammation and different therapies used. Small sample size may have affected the statistical results. A larger scale study could achieve more reliable results, also regarding the effect of a broader range of disease activity on QoL, which could also be assessed with disease-specific questionnaires in future studies. And finally, as the HLS-EU-Q16 is a self-assessment test, there is a potential for self-report bias by the participants. This also applies to the EQ-5D-5L and WPAI questionnaires.

In conclusion, this prospective and observational study evaluating HL of young Belgian adult CD patients demonstrates that in general, young adults who suffered from CD for at least 5 years have sufficient HL. However, the relationship between HL, QoL, and disease activity needs to be explored further to benefit patient management and well-being. Structured educational programs should be offered from the time of diagnosis of CD and provide for effective transition to self-care. We therefore recommend that national health care structures organize educational programs for children and adolescents afflicted with chronic diseases.

## Data Availability Statement

The raw data supporting the conclusions of this article will be made available by the authors, without undue reservation.

## Ethics Statement

The studies involving human participants were reviewed and approved by IRB ZNA Middelheim, Antwerp, Belgium. The patients/participants provided their written informed consent to participate in this study.

## Author Contributions

CC elaborated the protocol, conducted the study, collected and analyzed the data, statistics, and wrote the manuscript. PB and the scientific committee of the BIRD elaborated and approved the protocol. LW elaborated the protocol, collected and analyzed the data, statistics, contributed to the discussion and mentoring. RH coordinated the study on diabetes patients, contributed to the discussion and mentoring. GV initiated and wrote the protocol, coordinated the study and the writing process, contributed to the mentoring. All authors contributed to obtaining data, reviewed the protocol, the results, the intellectual content and discussion, and contributed to the review and approval of the manuscript.

## Conflict of Interest

The authors declare that the research was conducted in the absence of any commercial or financial relationships that could be construed as a potential conflict of interest.

## Abbreviations

AI: Activity Impairment
BELCRO: Belgian Crohn's Disease Registry
BESPGHAN: Belgian Society for Pediatric Gastroenterology, Hepatology and Nutrition
BIRD: Belgian IBD Research and Development
BMI: Body Mass Index
CD: Crohn's Disease
CDAI: Crohn's Disease Activity Index
CRF: Clinical Report Form
DM: Diabetes Mellitus
EC: Ethical Committee
EQ: EuroQol
FU: Follow-up
GH: General Health
HbA1c: Glycated hemoglobin
HL: Health Literacy
HLS-EU-Q: European Health Literacy Survey Questionnaire
IBD: Inflammatory Bowel Disease
IQR: Interquartile Range
IRB: Institutional Review Board
QoL: Quality of Life
UC: Ulcerative Colitis
VAS: Visual Analog Scale
WHO: World Health Organization
WPI: Work Productivity Impairment
WPAI: Work Productivity and Activity Impairment.
